# (Non)specific interaction patterns in families raising a child with disability or chronic illness: a latent profile analysis

**DOI:** 10.3389/fpsyg.2025.1555879

**Published:** 2025-04-23

**Authors:** Ariadna Łada-Maśko, Urszula Sajewicz-Radtke, Paweł Jurek, Michał Olech, Bartosz M. Radtke, Małgorzata Lipowska

**Affiliations:** ^1^Institute of Psychology, University of Gdańsk, Gdańsk, Poland; ^2^Laboratory of Psychological and Educational Tests, Gdańsk, Poland; ^3^Department of Psychology, Medical University of Gdańsk, Gdańsk, Poland

**Keywords:** child disability, siblings relationships, adolescents, family dynamics, adjustment challenges, intellectual disability, motor disability, diabetes

## Abstract

**Introduction:**

The presence of a child with a disability significantly affects family dynamics introducing new responsibilities and altering roles. However, research also highlights the positive outcomes, such as increased family cohesion and stronger bonds among family members. Siblings face unique challenges, including emotional burdens and adjustment difficulties, which are influenced by factors such as age, severity of the disability, and parental attitudes. While some siblings develop empathy and nurture traits, others struggle with internalizing and externalizing problems. This study investigated whether families exhibit distinct interaction patterns based on the presence and type of a child’s disability, considering the perspectives of healthy siblings and parents.

**Methods:**

The study comprised 179 dyads, each consisting of a healthy adolescent sibling of a child with a disability or chronic illness and one of their parents (*N* = 358). The sample included 49 families of children with diabetes, 49 with intellectual disabilities, 28 with motor disabilities, and 53 healthy children. To assess interaction patterns within families, the following measures were used: the Questionnaire of Relationships with Siblings to evaluate relationships between healthy adolescents and their siblings, the Parentification Questionnaire for Youth to measure emotional and instrumental parentification, the KidScreen-27 to assess quality of life, the Parental Attitude Scale-2 to assess parental attitudes, and the Family Rating Scales to evaluate family dynamics, including the structure and quality of family interactions. Latent Profile Analysis (LPA) was used to analyze data.

**Results:**

We identified two latent profiles: “Strained Families” profile, which featured lower-quality family interactions with parents displaying greater rejection, inconsistent, and overdemanding attitudes, and the “Resilient Families” profile reflected balanced functioning with greater cohesion and flexibility. The “Strained Families” profile was more prevalent (57%). No significant differences in profile frequencies were found across the groups.

**Conclusion:**

Both profiles included families with children with motor disabilities, intellectual disabilities, or diabetes and healthy children. Therefore, the results suggest that family interaction patterns varied independently of the presence of a child with a disability. Notably, 64% of the families with healthy children exhibited the “Strained Families” profile, marked by lower-quality interactions and family functioning, suggesting that such interactions are not exclusive to families of children with disabilities.

## 1 Introduction

Families of children facing a disability or chronic illness must navigate a complex reality that reshapes their daily lives, relationships, and overall functioning. The presence of a child with unique needs can significantly influence family dynamics, introduce challenges that disrupt existing routines, and require substantial adaptation from all members. These families frequently experience increased caregiving responsibilities, financial strain, social stigma, and emotional stress, which can have far-reaching implications for their individual and collective wellbeing (Aydogan and Kizildag, 2017; Cuzzocrea et al., 2013). Despite these challenges, many families discover opportunities for growth, resilience, and enriched relationships, highlighting both the challenges and opportunities within their experiences (Burke, 2004; Lee et al., 2015). Family interaction patterns are critical in determining a family’s ability to adapt to these challenges.

Interaction patterns within families play a central role in shaping the responses to the demands of a child’s disability. These patterns, characterized by how family members communicate, provide support, manage conflict, and bond, play a key role in determining a family’s adaptability and cohesion (Olson, 2011; Sekułowicz et al., 2022). From a systems theory perspective, the family operates as an interconnected unit in which each member’s behavior and wellbeing influence others (Masten and Monn, 2015). This perspective highlights several key principles that shape family interaction patterns, including feedback loops, homeostasis, and interdependence. Feedback loops, both positive and negative, regulate interactions by reinforcing or counteracting behavioral patterns within the family system, thereby influencing its stability and adaptability. Homeostasis refers to the family’s ability to maintain functional equilibrium despite external stressors, such as raising a child with a disability or chronic illness, which often necessitates adjustments in roles and responsibilities. Interdependence underscores the interconnected nature of family members, where changes in one individual’s wellbeing or behavior inevitably affect the entire system. Together, these principles provide a framework for understanding how families respond to challenges and reorganize their interactions to foster resilience and cohesion (Hooper, 2007; Murphy et al., 2017; Priest, 2021). However, a child’s disability can act as a stressor that disrupts stability, requiring families to develop new interaction strategies that balance the competing needs of the child, healthy siblings, and parents (Murphy et al., 2017; Walker et al., 2016). Understanding the perspectives of healthy siblings and parents is crucial because their experiences provide a comprehensive view of how families function under such circumstances.

Parental perspectives further illustrate the intricate dynamics of these families. Parents of children with disabilities or chronic illnesses often assume non-normative roles such as therapists, advocates, and educators for their children. These roles can foster personal growth, life wisdom, and a deepened sense of purpose as parents develop new skills and strengthen family bonds (Schippers et al., 2020; Hove et al., 2009). However, these benefits often coexist with intense stress arising from the demands of caregiving, financial pressures, and societal expectations (Parchomiuk, 2022; Pelentsov et al., 2016). Research has consistently demonstrated that parents’ wellbeing is intricately linked to their interactions with their children and the ability to maintain a balanced family environment (Totsika et al., 2014).

Parental stress is frequently reflected in distinct parenting styles that further shape family interactions. For instance, stressed parents may adopt overprotective behaviors, seeking to shield their child with a disability from further harm, but inadvertently limiting the child’s autonomy and reinforcing dependency. Alternatively, chronic stress may lead some parents to withdraw emotionally, reducing their capacity to engage meaningfully with both their child with a disability and their healthy siblings (Cuzzocrea et al., 2013; Walker et al., 2016) Such emotional withdrawal can result in diminished support and increased feelings of isolation among all family members, while overprotectiveness may contribute to heightened sibling rivalry or feelings of inadequacy in healthy siblings. Research has consistently shown that these stress-induced parenting styles can compromise family cohesion and adaptability, exacerbating challenges in managing the complex demands of caregiving (Jankowska et al., 2015; Lipowska et al., 2021; Totsika et al., 2014). Understanding the interplay between parental stress, parenting styles, and family interactions is therefore crucial for developing effective support strategies that address the needs of both the child with a disability and healthy siblings. While parents often assume primary caregiving roles, healthy siblings bear unique responsibilities and emotional challenges that impact their family experiences.

Research has highlighted that siblings of children with disabilities or chronic illnesses frequently encounter heightened caregiving duties, diminished parental attention, and increased psychological stress. These experiences can lead to resentment, loneliness, and guilt (Burke, 2004; Kurtuncu and Arslan, 2018). Siblings may struggle with the emotional weight of witnessing their siblings’ struggles, compounded by societal stigma and the expectation of maturing quickly (Mandleco and Webb, 2015; Takataya et al., 2019). However, many siblings report positive outcomes such as increased empathy, patience, and altruism, highlighting the complex nature of their experiences (Cuskelly and Gunn, 2003; Roper et al., 2014). Sibling relationships, a specific subset of family interactions, are particularly affected by the presence of a child with a disability or chronic illness. These relationships are often characterized by both closeness and conflict, with variations depending on factors such as age, gender, and type of disability (Burke, 2004; Kaminsky and Dewey, 2001). For instance, siblings of children with autism spectrum disorders may report less prosocial behavior and more competitive dynamics than siblings of children with Down syndrome (Roper et al., 2014). Despite these challenges, siblings often develop a profound sense of connection and pride in their roles, and a greater understanding of diversity and compassion (Mulroy et al., 2008; Pilowsky et al., 2004).

Interaction patterns within these families are influenced by numerous factors, including the type and severity of the child’s condition, family resources, and the sociocultural context. For example, families with a child with an intellectual disability may face different challenges and opportunities than those caring for a child with a chronic somatic illness such as diabetes. The level of behavioral problems exhibited by the child, the availability of external support, and the family’s coping strategies shape their interaction dynamics (Hastings and Petalas, 2014; Roper et al., 2014). Balanced levels of cohesion and adaptability, as described by Olson’s circumplex model, are critical for healthy family functioning. Families that achieve this balance are better equipped to manage stress and maintain supportive relationships (Olson, 2011; Olson et al., 1979).

The broader concept of family quality of life (FQoL) provides a valuable framework for examining interaction patterns within families facing the challenges associated with a child’s disability or chronic illness. FQoL encompasses the collective wellbeing of families, including their ability to meet individual and shared needs, maintain positive relationships, and achieve stability and satisfaction (Park et al., 2003). It is typically measured across multiple dimensions—family interactions, parenting, health and safety, and a general resources including emotional, social and emotional wellbeing– which together capture both the internal dynamics and external support structures of the family (Park et al., 2003; Brown et al., 2006). In families with a child facing a disability or chronic illness, FQoL is particularly relevant, as it not only reflects the direct impact of caregiving demands but also underscores how adaptive family interaction patterns can mitigate the negative effects of stress. While families with a child facing a disability or chronic illness often report lower levels of FQoL, the extent of this impact varies widely based on factors such as socioeconomic status, access to support services, and internal family dynamics (Brown et al., 2006). Studies suggest that fostering strong family relationships and providing adequate external resources can mitigate many of the negative effects of caregiver stress (Green, 2007; Zhang et al., 2015). By integrating the concept of FQoL with an analysis of family interaction patterns, we gain a more holistic understanding of how internal dynamics and external resources contribute to overall family functioning and wellbeing.

This study investigated the interaction patterns within families with a child facing a disability or chronic illness, with a particular focus on the perspectives of healthy siblings and parents. Using latent profile analysis, this study aims to identify distinct interaction patterns and explore how family members cope with the challenges posed by a child’s condition. Additionally, this study examines how these patterns vary depending on the type of disability or chronic illness, providing valuable insights for developing individualized family support interventions. Therefore, the following research questions are formulated:

1. Do healthy siblings perceive distinct family interaction patterns in families with a child facing a disability or chronic illness. If so, do these patterns vary by disability type?
2. Do parents report distinct family interaction patterns when raising a child with a disability or chronic illness? If so, do these patterns differ based on disability type?

## 2 Materials and methods

### 2.1 Participants and procedure

This study was conducted by the principles of the Declaration of Helsinki. Approval was granted by the Ethics Board for Research Projects of the Faculty of Social Sciences, University of Gdansk, Poland (decision no. 06/2022). The protocol of this study has been registered at: https://clinicaltrials.gov/, registration number: NCT06156124 and published (Łada-Maśko et al., 2024).

This cross-sectional case–control study included 179 dyads, each comprising a healthy adolescent sibling of a child with a disability or chronic illness and one of their parents (*N* = 358). Among the adolescents (*M_age_* = 16.68, *SD* = 0.67), there were 96 girls (54%; *M_age_* = 16.67, *SD* = 0.68), and 83 boys (46%; *M_age_* = 16.70, *SD* = 0.68). Most participating parents were mothers (*N* = 156, 87%). The sample comprised 49 families with children diagnosed with diabetes (27%), 49 with intellectual disabilities (27%) 28 with motor disabilities (16%), and 53 healthy children (30%). Sixteen families resided in rural areas (9%), 13 families (7%) were from small towns with populations of up to 20,000, 49 families (27%) lived in towns with populations between 20,000 and 100,000, and the remaining 101 families (56%) were from cities with populations exceeding 100,000.

Participants were recruited from educational institutions, psychological and educational counseling centers, hospitals, therapeutic centers, and social media advertisements. Data collection was conducted through two methods: (1) an online version of the questionnaires battery, completed via participants’ personal electronic devices (e.g., mobile phones, laptops, tablets); and (2) a paper-and-pencil version administrated by the project principal investigator in participants’ everyday settings (e.g., schools).

The inclusion criteria for the group with a disabled sibling were as follows: participants were required to be in late adolescence (16–18 years), have no disabilities or disorders, and have a sibling with, depending on the group, intellectual disability, motor disability, or chronic somatic disease (diabetes). Additionally, the participation of one parent in the study was mandatory. For the control group (siblings without disabilities), the inclusion criteria were identical in terms of age and parental participation; however, the participants were required to have healthy siblings without any disabilities. The exclusion criteria for the group with a disabled sibling included cases in which the sibling had multiple disabilities (e.g., intellectual and motor disabilities) in order to ensure homogeneity across groups and to facilitate the specific characteristics unique to each type of disability. Adolescents with disabilities, disorders, or chronic diseases were excluded.

### 2.2 Measures

This study examined family interaction patterns encompassing sibling relations, parental attitudes, quality of family life, family dynamics, and parentification. Several questionnaires were administered to assess these dimensions.

#### 2.2.1 Siblings’ relations

The *Questionnaire of Relationships with Siblings* (Lewandowska-Walter et al., 2016) was used to assess the quality of relationships between healthy adolescents and their siblings. This questionnaire consists of 20 items divided into three subscales: (1) *Cohesion* (8 items, e.g., “I can count on the support of my siblings when I am in a difficult situation”), (2) *Communication* (6 items, e.g., “I talk to my siblings about things that are important to me”), and (3) *Rivalry* (6 items, e.g., “The siblings are jealous of me”). The *Cohesion* subscale measures the strength of bonds between siblings, ranging from strong to no bonds. The *Communication* subscale evaluates the quality of communication between siblings on a continuum from effective to problematic. The *Rivalry* subscale assesses sibling competition, ranging from positive (motivating competition) to angry (intense competition for parental attention). Participants rated the frequency of their described feelings and behaviors toward their siblings on a 5-point Likert scale from 1 (never) to 5 (very often). The subscale scores were calculated by summing the responses to the items within each subscale. Cronbach’s *α* values in this study were as follows: 0.89 for *Cohesion*, 0.87 for *Communication,* and 0.74 for *Rivalry*.

#### 2.2.2 Parental attitudes

The *Parental Attitude Scale-2* (SPR-2; Plopa, 2012) was used to assess the adolescents’ perceptions of their parental attitudes. This measure evaluates five distinct parental attitudes: *Acceptance-Rejection, Overdemanding, Autonomy, Inconsistency, and Overprotective*. The *Acceptance-Rejection* attitude reflects the degree to which a parent accepts their child. Low scores indicated insensitive and rejecting behaviors, whereas high scores reflected accepting, supportive, and sensitive parental approaches. An *Overdemanding* attitude reflects the level of parental expectations and rigidity. High scores in this domain suggest a strict and inflexible approach characterized by a lack of understanding of adolescents’ autonomy and rigid enforcement of rules. The *Autonomy* attitude represents a flexible and developmentally appropriate parenting style in which the child is treated as an individual capable of independent thought and decision-making. This *Inconsistency* attitude captures the fluctuating parental behavior that varies with the parent’s mood, wellbeing, or personal circumstances. An *Overprotective* attitude measures the tendency to perceive the child as helpless, dependent, and needing constant control, reflecting a lack of recognition of the adolescent’s growing need for autonomy. The SPR-2 consists of 45 items assessed separately for mothers and fathers. Participants responded on a 5-point Likert scale from 1 (not true at all) to 5 (entirely true), indicating how each statement corresponded to their parent’s behavior. Higher scores signified a stronger intensity of the given parental attitude perceived by adolescents. Cronbach’s *α* values in this study were as follows: 0.97 and 0.97 for *Acceptance-Rejection* parental attitude (for mothers and fathers respectively), 0.90 and 0.91 for *Overdemanding*, 0.95 and 0.94 for *Autonomy*, 0.91 and 0.92 for *Inconsistency*, and 0.86 and 0.86 for *Overprotective.*

#### 2.2.3 Quality of family life

The Polish adaptation of the *KidScreen-27* (Mazur et al., 2008) was used in this study to assess the quality of life regarding autonomy and parental relationships from the perspective of healthy siblings. This health-related quality of life questionnaire, developed by the KIDSCREEN Research Group across 13 countries (Robitail et al., 2007), consists of 27 items. Adolescents responded to each question on a 5-point scale (1 = never to 5 = always). The *KidScreen-27* measures five dimensions of quality of life: (1) physical wellbeing, (2) psychological wellbeing, (3) parent relationships and autonomy, (4) social support and peers, and (5) school environment. In this study, only the *parent relationships and autonomy* dimensions were used. Cronbach’s *α* values in this study for this subscale was 0.84.

#### 2.2.4 Family dynamics

*Family Rating Scales* (SOR; Margasiński, 2009, 2015) were used to comprehensively assess family dynamics, including the structure and quality of family interactions. SOR is the Polish adaptation of the American *FACES IV* (Flexibility and Cohesion Scales) questionnaire, developed initially by Olson (2011). The SOR consists of 62 items organized into eight subscales. These include two balanced scales: *Balanced Cohesion* and *Balanced Flexibility,* four unbalanced scales (which assess dysfunction in the dimensions of cohesion and flexibility): *Disengaged* and *Enmeshed* for cohesion, and *Rigid* and *Chaotic* for flexibility; and two evaluative scales*: Communication* and *Family Life Satisfaction*. Each item is rated on a five-point scale ranging from 1 to 5. The questionnaire allows the calculation of individual subscale scores and composite indicators, such as the Balanced/Unbalanced Ratio Score, which provides an overall measure of family functioning. This ratio indicates whether the family system is balanced or unbalanced, with a score >1 indicating a balanced system and a score lower than 1 suggesting an unbalanced system. In the present study we used subscale scores to better capture the distinct dimensions of family interactions relevant to our research objectives. Cronbach’s *α* values in this study were as follows: 0.73 for *Balanced Cohesion*, 0.62 for *Balanced Flexibility*, 0.73 for *Disengaged*, 0.62 for *Enmeshed,* 0.64 for *Rigid*, 0.70 for *Chaotic*, 0.91 for *Communication*, 0.93 for *Family Life Satisfaction.*

#### 2.2.5 Parentification

The *Parentification Questionnaire for Youth* (PQY; Borchet et al., 2020) was used in this study. This self-report questionnaire captures the multidimensional nature of parentification. The PQY consists of 26 items, with participants rating each statement on a 5-point Likert scale from 1 (never true) to 5 (always true). The questionnaire is divided into four primary subscales: (1) *Emotional Parentification toward Parents,* (2) *Instrumental Parentification toward Parents*, (3) *Sense of Injustice,* and (4) *Satisfaction with the Role*, as well as two additional subscales for adolescents with siblings: (5) *Instrumental Parentification toward Siblings* and (6) *Emotional Parentification toward Siblings.* The PQY does not provide a total score. Instead, the scores for each subscale are calculated as the mean of the responses to the items within that subscale. Cronbach’s α values in this study were as follows: 0.71 for *Emotional Parentification toward Parents,* 0.59 for *Instrumental Parentification toward Parents*, 0.85 for *Sense of Injustice,* 0.71 for *Satisfaction with the Role*, 0.79 for *Instrumental Parentification toward Siblings,* and 0.84 for *Emotional Parentification toward Siblings.*

### 2.3 Statistical analysis

To address the research question of whether there is a specific pattern of family interactions in families with a child with a disability or chronic illness based on the perspectives of healthy siblings and parents, we employed a two-step analytical approach. First, we fitted a Latent Profile Analysis (LPA) model to identify distinct interaction patterns. Second, we examined the frequency of these latent profiles in families with children with specific disabilities or chronic illnesses.

LPA is a model-based technique that identifies latent subgroups by estimating probabilities of class membership rather than relying on arbitrary distance measures, as in traditional cluster analysis (Woo et al., 2024). Unlike cluster analysis, LPA assumes data arise from a mixture of multivariate distributions and accounts for classification uncertainty. It also provides fit indices to compare model solutions and accommodates measurement error, making it a more robust and flexible method (Hagenaars and McCutcheon, 2002; Lazarsfeld, 1950). Given the complexity of family dynamics in households with a child with a disability or chronic illness, LPA is particularly suitable for identifying meaningful interaction patterns. Following Weller et al. (2020), analyses were conducted using the tidyLPA package (Rosenberg et al., 2018) in the R programming environment (R Core Team, 2024).

LPA was based on indicators measured on a 1–5 scale, calculated as the arithmetic mean of responses to items comprising each observed variable. Two exceptions were the Flexibility and Cohesion subscales of the Family Rating Scales, which were linearly transformed to fall within the range of 1–5. This adjustment was necessary to ensure comparability with other variables. The LPA model employs the EEE variant (equal variance and covariance). This model assumes that the latent profiles differ in the means of the observed variables but have equal variances within the profiles. Additionally, it imposes the constraint that the covariance matrix is diagonal, implying no correlation among the observed variables within each latent profile. This structure ensures that the differences between profiles are driven by mean differences rather than by the covariance structure of the variables.

To select the optimal number of latent profiles, we followed the best practice guidelines outlined by Weller, Bowen, and Faubert (2020). We considered models with 2–4 profiles, guided by the following criteria: (a) each profile was required to include at least 5% of the sample and a minimum of 50 cases; (b) model fit was evaluated using standard information criteria, including the Bayesian Information Criterion (BIC) and the Akaike Information Criterion (AIC); and (c) entropy was assessed to ensure a precise classification of cases into latent profiles.

Although Nylund-Gibson and Choi (2018) recommend a sample size of at least 300 cases for LPA, achieving this threshold was not feasible due to the challenges of recruiting entire families rather than individuals from a hard-to-reach population. However, methodological literature emphasizes that the adequacy of sample size in LPA depends not only on absolute numbers but also on the separation between profiles, the number of indicators, and entropy values (Berlin et al., 2014). Given the relatively distinct profiles identified in our study and acceptable entropy levels, we believe our sample is sufficient to detect meaningful differences, while recognizing the inherent limitations. Additionally, we acknowledge more flexible guidelines regarding minimum sample size (e.g., Nagin, 2005), highlighting that no universal standard exists for LPA and that decisions must consider the specific research context.

After identifying the optimal number of profiles, we computed the frequency of families with children having specific disabilities or chronic illnesses within each latent profile. We used a chi-squared test to examine whether latent profile membership differed across family groups defined by the child’s condition.

This two-step analytical approach allowed us to identify distinct patterns of family interactions and to evaluate their prevalence in families with specific disabilities or chronic illnesses.

## 3 Results

### 3.1 Latent profile analysis

Table 1 presents the descriptive statistics for the variables included in the Latent Profile Analysis (LPA) after linear transformation to a range of 1–5, including means and standard deviations. Additionally, it reports skewness and kurtosis values to examine potential deviations from normality, following the guidelines of Curran et al. (1996). Based on the established thresholds (skewness <2.0, kurtosis <7.0), the distribution of all variables did not indicate substantial departures from normality. These findings suggest that the data meet the assumptions necessary for conducting LPA without significant concerns regarding nonnormality.

**Table 1:** Descriptive statistics and normality indices for variables used in latent profile analysis.

Questionnaire/variable	Min.	Max.	*M*	*SD*	Skewness	Kurtosis
Questionnaire of Relationships with Siblings						
Cohesion	1.00	5.00	3.52	0.88	−0.41	−0.39
Communication	1.00	4.83	3.06	0.92	−0.18	−0.74
Rivalry	1.17	5.00	3.90	0.72	−0.47	0.53
Parentification Questionnaire for Youth						
Instrumental Parentification toward Siblings	1.00	4.75	2.55	0.95	0.16	−0.76
Emotional Parentification toward Siblings	1.00	5.00	2.68	0.89	0.07	−0.37
Instrumental Parentification toward Parents	1.00	4.25	1.94	0.62	0.86	1.04
Emotional Parentification toward Parents	1.00	5.00	2.82	0.80	0.03	−0.19
Satisfaction with the Role	1.50	5.00	3.48	0.75	−0.09	−0.57
Sense of Injustice	1.00	4.40	2.21	0.87	0.35	−0.88
KidScreen-27						
Autonomy and Parent Relations	1.71	5.00	3.43	0.71	−0.05	−0.61
Parental Attitude Scale - 2						
Acceptance-Rejection (mother)	1.00	5.00	3.54	1.26	−0.60	−0.94
Autonomy (mother)	1.00	5.00	3.44	1.15	−0.56	−0.87
Overprotective (mother)	1.11	5.00	3.20	0.89	−0.01	−0.55
Overdemanding (mother)	1.00	4.78	2.87	0.96	−0.11	−0.89
Inconsistent (mother)	1.00	5.00	2.67	1.08	0.40	−0.87
Acceptance-Rejection (father)	1.00	5.00	3.66	1.15	−0.70	−0.54
Autonomy (father)	1.00	5.00	3.51	1.09	−0.62	−0.63
Overprotective (father)	1.00	5.00	3.34	0.87	−0.20	−0.25
Overdemanding (father)	1.00	5.00	2.80	1.01	0.03	−0.95
Inconsistent (father)	1.00	5.00	2.61	1.08	0.43	−0.84
Family Rating Scales						
Balanced cohesion	2.00	5.00	4.15	0.62	−0.80	0.10
Balanced flexibility	1.86	5.00	3.73	0.61	−0.51	−0.05
Disengagement	1.00	3.71	1.91	0.68	0.64	−0.49
Enmeshment	1.00	3.86	2.00	0.64	0.34	−0.45
Rigidity	1.14	4.57	2.75	0.70	0.34	−0.59
Chaos	1.00	4.14	2.19	0.73	0.55	−0.21
Family Communication	1.60	5.00	4.11	0.70	−0.94	0.65
Family Life Satisfaction	1.20	5.00	4.05	0.75	−1.02	0.76
Flexibility	1.48	4.72	2.80	0.76	0.53	−0.34
Cohesion	1.48	3.70	2.15	0.37	0.70	0.97

**Figure 1:**
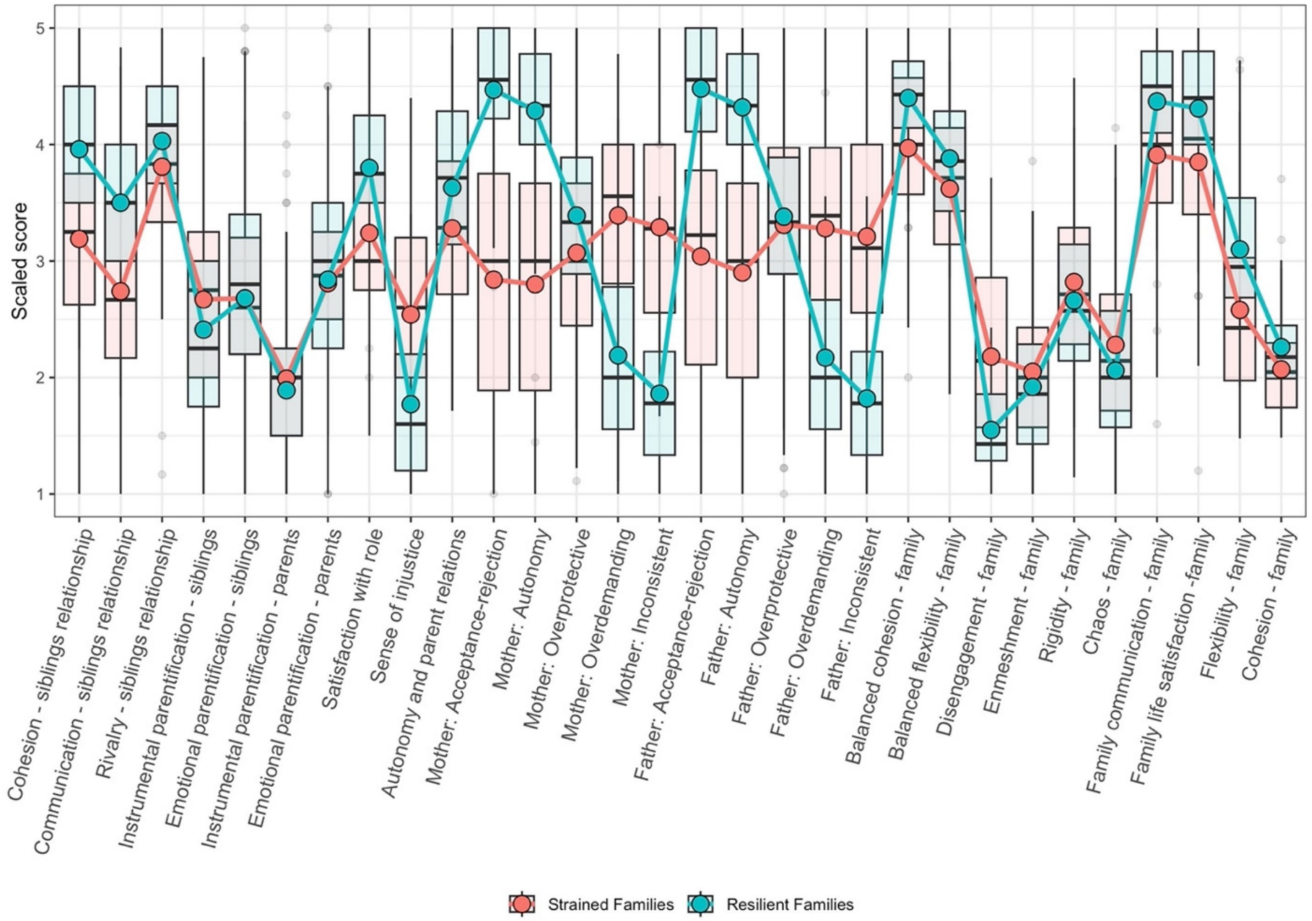
Distributions of key variables across identified family profiles: Strained families vs. Resilient families.

Table 2 summarizes the fit indices and selected characteristics of the LPA models for the two to four profiles. All analyzed models demonstrated a good model fit according to the evaluated criteria. Consequently, the decisive factor in the model selection was the number of cases assigned to each profile. Table 3 presents the descriptive statistics for the two identified latent profiles. In addition to the mean and standard deviation for each profile, Table 3 includes the mean difference between groups (*Δ* mean) along with 95% confidence intervals. Table 3 also presents the results of pairwise comparisons across scales using the Student’s *t*-test and reports the effect sizes as Cohen’s d. Furthermore, to account for multiple comparisons, we applied Holm’s correction, which sequentially adjusts *p*-values to control the family-wise error rate. Figure 1 provides a graphical visualization of the comparison between the latent profiles, illustrating their distinguishing characteristics.

**Table 2:** Summary of fit indices and selected characteristics of LPA models for 2–4 profiles.

No. profiles	AIC	BIC	Entropy	Probability		Percent of sample		*N*	
				Min.	Max.	Min.	Max.	Min	Max
2	8150.82	9827.38	0.96	0.99	1.00	43.02	56.98	77	102
3	8067.69	9843.07	0.98	0.99	1.00	11.73	48.60	21	87
4	8022.00	9896.19	0.98	0.99	1.00	11.73	38.55	21	69

**Table 3:** Descriptive statistics, profiles comparisons, and effect sizes for identified latent profiles.

Questionnaire/variable	Profile 1 Strained families		Profile 2 Resilient families		*t*	*p* adjusted		Δ mean [95% CI]	Cohen d	Magnitude
	*M*	*SD*	*M*	*SD*						
Questionnaire of Relationships with Siblings										
Cohesion	25.51	7.02	31.66	5.27	−6.7	< 0.01**		−6.15 [−7.96, −4.34]	−0.991	Large
Communication	16.42	5.51	20.99	4.32	−6.21	< 0.01**		−4.57 [−6.02, −3.12]	−0.922	Large
Rivalry	22.86	4.20	24.17	4.34	−2.02	0.06		−1.31 [−2.58, −0.03]	−0.306	Small
Parentification Questionnaire for Youth										
Instrumental Parentification toward Parents	1.99	0.97	1.89	0.91	1.05	0.33		0.10 [−0.08, 0.28]	0.156	Negligible
Emotional Parentification toward Parents	2.81	0.93	2.84	0.85	−0.2	0.87		−0.03 [−0.27, 0.22]	−0.030	Negligible
Satisfaction with the Role	3.24	0.67	3.80	0.55	−5.4	<0.01**		−0.56 [−0.77, −0.36]	−0.809	Large
Sense of Injustice	2.54	0.77	1.77	0.84	6.7	<0.01**		0.77 [0.54, 1.00]	0.998	Large
Instrumental Parentification toward Siblings	2.67	0.74	2.41	0.65	1.85	0.08		0.26 [−0.02, 0.54]	0.278	Small
Emotional Parentification toward Siblings	2.68	0.85	2.68	0.69	−0.02	0.99		0.00 [−0.27, 0.26]	−0.002	Negligible
KidScreen-27										
Autonomy and Parent Relations	22.95	4.99	25.40	4.65	−3.38	0.01**		−2.45 [−3.88, −1.02]	−0.508	Moderate
Parental Attitude Scale - 2										
Acceptance-Rejection (mother)	25.59	10.44	40.19	5.84	−11.88	<0.01**		−14.6 [−17.03, −12.18]	−1.727	Large
Autonomy (mother)	25.23	9.33	38.60	5.80	−11.77	<0.01**		−13.37 [−15.61, −11.13]	−1.722	Large
Overprotective (mother)	27.60	8.20	30.47	7.53	−2.43	0.02*		−2.87 [−5.20, −0.54]	−0.365	Small
Overdemanding (mother)	30.47	6.77	19.74	6.93	10.36	<0.01**		10.73 [8.69, 12.78]	1.567	Large
Inconsistent (mother)	29.58	8.29	16.78	5.87	12.09	<0.01**		12.80 [10.71, 14.89]	1.781	Large
Acceptance-Rejection (father)	27.32	9.99	40.36	4.65	−11.62	<0.01**		−13.04 [−15.26, −10.82]	−1.673	Large
Autonomy (father)	26.14	9.08	38.90	4.70	−12.2	<0.01**		−12.76 [−14.82, −10.69]	−1.766	Large
Overprotective (father)	29.81	8.31	30.44	7.29	−0.54	0.63		−0.63 [−2.94, 1.68]	−0.080	Negligible
Overdemanding (father)	29.56	8.10	19.49	6.98	8.91	<0.01**		10.07 [7.84, 12.30]	1.331	Large
Inconsistent (father)	28.89	8.71	16.42	5.49	11.71	< 0.01**		12.47 [10.37, 14.58]	1.714	Large
Family Rating Scales										
Balanced Cohesion	27.78	4.74	30.78	3.09	−5.11	<0.01**		−3.00 [−4.15, −1.84]	−0.749	Moderate
Balanced Flexibility	25.35	4.56	27.13	3.68	−2.88	<0.01**		−1.78 [−2.99, −0.56]	−0.429	Small
Disengagement	15.25	5.08	10.82	2.73	7.5	<0.01**		4.43 [3.27, 5.60]	1.088	Large
Enmeshment	14.37	4.42	13.43	4.48	1.4	0.19		0.94 [−0.38, 2.27]	0.212	Small
Rigidity	19.71	4.97	18.65	4.68	1.46	0.18		1.06 [−0.38, 2.49]	0.219	Small
Chaos	15.98	5.20	14.39	4.91	2.09	0.05		1.59 [0.09, 3.09]	0.314	Small
Family Communication	39.11	7.26	43.73	5.60	−4.81	<0.01**		−4.62 [−6.52, −2.72]	−0.712	Moderate
Family Life Satisfaction	38.49	8.11	43.06	5.56	−4.47	<0.01**		−4.57 [−6.59, −2.56]	−0.658	Moderate
Flexibility	0.92	0.62	1.33	0.65	−4.29	<0.01**		−0.41 [−0.60, −0.22]	−0.650	Moderate
Cohesion	0.90	0.37	1.10	0.38	−3.48	<0.01**		−0.20 [−0.31, −0.09]	−0.527	Moderate

**p < 0.01, *p < 0.05.

This analysis revealed two distinct family interaction profiles. Lower scores on the positive dimensions of family interactions, such as cohesion, communication, and flexibility, among siblings and between parents and children characterize the first profile, which can be considered as “Strained Families” profile. Furthermore, parents in these families tended to exhibit less overprotective and inconsistent behaviors, with lower levels of autonomy and acceptance of parental attitudes. Consequently, these families often experience greater disengagement, reducing family cohesion and overall satisfaction.

By contrast, the second profile, which could be described as “Resilient Families” profile, represented families with significantly higher scores on the positive interaction dimensions. These families demonstrate strong cohesion, effective communication, and greater flexibility in their relationships among siblings, parents, and children. The siblings’ emotional identification was notably lower in this group. Parents in these families are more likely to support their children’s autonomy and exhibit acceptance, balance, and consistent parental attitudes. Overall, families in this profile were characterized by higher adaptability, cohesion, and satisfaction.

In summary, the first profile reflects families facing greater challenges in their interaction patterns, which may hinder overall family functioning, whereas the second profile highlights families with healthier and more supportive relationships.

### 3.2 Association between child’s condition and family interaction profiles

Table 4 presents the frequency of families with children with specific disabilities or chronic illnesses across the identified latent profiles. The chi-square test (*χ*^2^ = 7.55, df = 3, *p* = 0.06) indicated that the observed differences in frequencies across subgroups were not statistically significant. This suggests that there are no significant differences in the distribution of families assigned to “Strained Families” and “Resilient Families” profiles within the four family groups. Therefore, the presence of a child with a disability or chronic illness did not significantly predict a specific family interaction profile.

**Table 4:** Frequencies of families with children with specific disabilities or chronic illness across latent profiles.

Family	Total	Strained families		Resilient families	
		*n*	%	*n*	%
With a child with diabetes	49	23	47	26	53
With a child with intellectual disability	49	33	67	16	33
With a child with motor disability	28	12	43	16	57
With children without disabilities or chronic illnesses	53	34	64	19	36
Total	179	102	57	72	43

## 4 Discussion

This study explored the interaction patterns within families raising children with disabilities or chronic illness, focusing on the perspectives of healthy adolescent siblings and parents. Using Latent Profile Analysis (LPA), we identified two distinct profiles: one reflecting lower-quality interactions (“Strained Families”) and the other characterized by balanced family functioning (“Resilient Families”). Our findings revealed significant differences between these profiles across most of the variables examined, including the quality of sibling relationships, healthy adolescents’ perceptions of their relationships with their parents, and their quality of life related to autonomy and parent–child connections. Furthermore, parental assessments of family functioning revealed significant differences in family system dynamics, with families in the first profile showing lower cohesion and higher rigidity than those in the second profile, demonstrating greater flexibility and balanced dynamics.

The “Strained Families” profile identified in this study seems to characterize families with fewer resources. From the perspective of healthy adolescents in these families, sibling relationships are characterized by lower cohesion, poorer communication quality, and lower levels of rivalry. We also observed a tendency toward higher levels of instrumental parentification directed toward siblings, although this did not reach statistical significance. Interestingly, no differences were observed in emotional parentification. Our findings align with previous studies on the impact of limited family resources on sibling and parentification roles. For instance, parentification, in which children assume adult-like responsibilities, can significantly affect family relationships and individual development (Borchet et al., 2016; Masiran et al., 2023). Additionally, research has highlighted that parentification can lead to stronger sibling bonds and promote empathy, understanding, and acceptance among siblings (Dariotis et al., 2023). However, our finding of no significant differences in emotional parentification contrasts with other studies, suggesting that parentification can negatively affect parentified children (Levante et al., 2022; Sharpe, 2024). This discrepancy may be explained by contextual factors, such as family coping strategies or cultural norms, which can moderate the impact of parentification on family relationships (Połomski et al., 2021). Families with adaptive coping mechanisms or those from cultures that emphasize intergenerational support may experience less emotional strain, as the parentified child may perceive their role as more normative or manageable. Additionally, family resources, such as external support networks, could alleviate the emotional burdens typically associated with parentification, highlighting the importance of considering these contextual factors in future research. Adolescents assigned to the “Strained Families” profile reported higher dissatisfaction with family roles and a heightened sense of injustice, likely due to unbalanced expectations and lack of support (Bi et al., 2018; Stapley et al., 2021). For instance, limited resources can heighten competition among family members for attention and support, leading to feelings of unfairness, particularly if some siblings are perceived as receiving more attention or care. Additionally, parental behaviors such as inconsistent discipline, emotional unavailability, or an over-reliance on certain family members for support may exacerbate feelings of dissatisfaction. These dynamics can create an environment where adolescents feel their needs are overlooked or undervalued, intensifying their sense of injustice (Burke, 2004; Takataya et al., 2019; Vermaes et al., 2012). Parents’ assessments of family functioning reflect similar negative patterns, with families exhibiting lower levels of balance and flexibility along with higher levels of rigidity, chaos, and disengagement, contributing to decreased overall family life satisfaction (Olson, 2011; Olson et al., 1979). Thus, the interaction patterns observed in the families assigned to “Strained Families” profile indicate numerous challenges both within the sibling subsystem and in parent–child relationships, as well as in family functioning as a system.

Interestingly, one of the most important findings of this study was that 64% of the families with healthy children were classified into the “Strained Families” profile, which was characterized by lower-quality interaction patterns. This result is particularly noteworthy because it suggests that even families without a child with a disability or chronic illness can experience significant challenges within their family dynamics, leading to poor-quality relationships. Previous research has indicated that families with healthy children may encounter stressors stemming from external or internal factors, such as economic hardship, parental mental health issues, or familial conflicts, which can negatively affect family functioning (Mphaphuli, 2023; Neppl et al., 2016). Moreover, studies on family resilience emphasize that family functioning is shaped not only by the presence of disability but also by factors such as parental stress, communication patterns, and sibling interactions (Barroso et al., 2018; Cheng et al., 2024; Lewandowski et al., 2010; Priego-Ojeda and Rusu, 2023). These findings suggest that the dynamics observed in the lower-quality interaction profile may reflect broader stressors that affect the family system as a whole, even in the absence of a disabled child. This finding highlights the complexity of family life and the importance of adopting a holistic approach to assess family wellbeing.

By contrast, the “Resilient Families” profile appeared to represent families with more resources to cope with challenges. Sibling relationships in these families were characterized by higher cohesion and better communication quality. Adolescents in these families reported greater satisfaction with their family roles and a significantly lower sense of injustice. Higher levels of acceptance, autonomy, and overprotectiveness marked parental attitudes toward these families. Both mothers and fathers in these families demonstrated significantly lower demanding and inconsistent parenting levels than those in the first profile. From the perspective of the parents, these families exhibited stronger cohesion, more effective communication, and greater flexibility, reflecting more balanced family functioning. These results suggest that families assigned to this profile experience healthier relationships among family members and more balanced family dynamics. Research supports these findings, indicating that families with more emotional, financial, or social resources tend to have healthier relationships with each other. For example, families with more financial resources and higher parental education often experience less conflict and better communication (Berger and Font, 2015; Duncan et al., 2010; Grevenstein et al., 2019). Additionally, studies show that parental autonomy support, which includes respecting children’s independence and providing appropriate guidance, fosters better family relationships and adolescent satisfaction (Bülow et al., 2022). Furthermore, research suggests that higher levels of overprotection in certain families can lead to positive and negative outcomes (Arslan et al., 2023). Although overprotective parents may provide more security and support to their children, they may also hinder their autonomy, leading to difficulties in developing their independence (Arslan et al., 2023; Flamant et al., 2022). In this case, the overprotectiveness observed in the second profile may contribute to a more controlled environment, potentially reducing adolescent dissatisfaction by ensuring consistent support. However, it could also limit adolescents opportunities for autonomy. Moreover, the increased family cohesion and communication quality in these families align with the findings that strong family cohesion and effective communication positively correlate with better psychological outcomes for adolescents (Fosco and Lydon-Staley, 2020; Olson, 2011). The greater flexibility and balanced family functioning reported in these families are consistent with the research on family systems theory, which emphasizes the importance of adaptability in family dynamics to promote healthy relationships (Deng et al., 2022; van Eickels et al., 2022; Uruk et al., 2007). Therefore, the positive family dynamics found in the second profile reflect a more resilient family system capable of navigating challenges in a balanced and supportive manner.

Furthermore, this study aimed to explore whether there is a specific interaction pattern within families raising a child with a disability or chronic illness, particularly from the perspective of healthy siblings and parents, and whether this pattern differs depending on the type of disability. Our results indicate that no clear pattern of interaction emerged based on disability type. Families, both with children with disabilities and healthy children were represented in both profiles. Moreover, the type of disability did not appear to differentiate the profiles, as families with children diagnosed with motor disabilities, intellectual disabilities, and chronic conditions such as diabetes were present in both groups. These findings contradict studies suggesting that different types of disabilities can lead to distinct interaction patterns and family stressors (Guralnick et al., 2008; Murphy et al., 2017; Walker et al., 2016). Other research have also emphasized the unique challenges that families face depending on the nature of their disability, particularly when long-term care or ongoing medical management is required (Cuzzocrea et al., 2013; Kaminsky and Dewey, 2001; Langley et al., 2021; Roper et al., 2014; Vermaes et al., 2012).

The finding that the type of disability did not significantly affect interaction patterns suggest that broader family dynamics, rather than the specific nature of the child’s condition, play a more decisive role in shaping family interactions. Although no significant differences were identified in the distribution of families across profiles, the observed variations suggest potential trends that warrant further consideration. Previous research indicates that factors such as the severity of the condition, behavioral challenges, family resources, and sociocultural context influence family functioning (Hastings and Petalas, 2014; Roper et al., 2014). The higher proportion of families with children with intellectual disabilities in “Strained Families” profile may reflect unique challenges associated with raising a child with cognitive impairments. For example, families of children with intellectual disabilities may face different demands than those managing a chronic somatic illness, yet both groups must navigate stress, external support availability, and coping strategies. Similarly, the distribution of families with healthy children suggests that lower-quality interaction patterns are not exclusive to families of children with disabilities. While these differences may not be definitive, they point to meaningful patterns in how families adapt to their circumstances. Future research with a larger and more diverse sample could clarify whether these trends reflect systematic differences in family functioning or result from other contextual factors.

The consistency of interaction patterns across different disability types may indicate that these overarching factors, rather than the diagnosis itself, are key determinants of family dynamics. Additionally, Olson’s circumplex model (Olson, 2011; Olson et al., 1979) emphasizes the importance of balanced cohesion and adaptability for healthy family functioning. Families that maintain this balance, regardless of their child’s specific diagnosis, may be more resilient in managing caregiving challenges.

## 5 Limitations

This study has several limitations that should be considered when interpreting the findings. First, it used a cross-sectional design that offered only a momentary view of family dynamics simultaneously. A longitudinal approach would provide greater value in examining how these dynamics evolve, particularly as children with disabilities or chronic illnesses grow older, and in understanding if and how family profiles shift across different developmental stages. Additionally, it would be valuable to explore how family dynamics change at various stages of the disability/illness trajectory, from searching for a diagnosis to receiving it and managing the condition thereafter. Understanding these transitions could provide deeper insight into the evolving nature of family interactions and how families cope with the challenges associated with chronic illness or disability at different stages.

Another limitation of the study is that the Cronbach’s *α* values for some measures were relatively low, including the Balanced Cohesion, Enmeshed, and Rigid subscales from the SOR, as well as the Instrumental Parentification toward Parents from the PQY. These low values may affect the measurement validity of these scales and should be taken into account when interpreting the results.

Additionally, the study was conducted in Poland, which raises concerns regarding the cultural context in which the findings were obtained. Family interaction patterns vary significantly across cultures and are influenced by distinct caregiving practices, norms, and values (Lansford, 2024; Park et al., 2022; Pastorelli et al., 2016; Sharma et al., 2022). Future research should explore whether similar family interaction profiles emerge in other countries, particularly in cultures with varying levels of collectivism, social support structures, and healthcare accessibility, while also exploring how family dynamics in families of children with disabilities differ across cultural contexts and identifying which aspects are culturally specific and which are universal.

Another limitation pertains to the generalizability of the findings due to sample characteristics. This study focused on a specific age range of siblings and was geographically limited to Poland. These factors may affect the applicability of the results to broader populations. To enhance the external validity of future studies, a more diverse sample, including families from different socioeconomic backgrounds and regions, would be beneficial.

## 6 Implications and future research

The findings of this study have important implications for clinical practice and future research. One key implication is the potential to develop targeted family interventions based on the specific interaction profiles identified in this study. For instance, families experiencing lower-quality interactions may benefit from family therapy, psychoeducational programs on stress management, and support groups to improve communication and coping strategies. Meanwhile, families with better-functioning dynamics might find preventive interventions, such as resilience-building programs, parenting workshops, or guidance on maintaining balanced family roles, more beneficial. Differentiating interventions based on family needs may enhance their effectiveness, leading to better long-term outcomes for both parents and children. Future studies could explore whether tailored interventions designed to address the unique needs of families based on their identified profiles might be more effective in improving family dynamics and enhancing the wellbeing of all family members, including parents and siblings (Desautels et al., 2020; Park et al., 2018). Additionally, future research could examine how family interaction patterns impact siblings’ and parents’ wellbeing, mental health, and overall adjustment. As previous research suggests, adolescent siblings of children with disabilities are at a higher risk of developing both internalizing disorders, such as depression and anxiety, and externalizing behaviors, such as aggression or conduct problems (Burke, 2004; Caliendo et al., 2020; Stephenson et al., 2017). Similarly, parents of children with disabilities are more vulnerable to psychological distress, particularly anxiety and depressive disorders (Arzeen et al., 2020; Scherer et al., 2019). Given these findings, future studies should further investigate the mechanisms through which family interaction patterns contribute to these mental health risks and identify protective factors that could mitigate their impact.

A particularly valuable direction for future research could also involve cross-cultural comparisons. Examining how family interaction patterns vary across different cultural contexts and how cultural attitudes toward disability or chronic illness influence family dynamics would provide important insights into the universality or cultural specificity of these patterns. Understanding how different societies approach caregiving, family roles, and disability could lead to more culturally informed and effective interventions.

Future research could also benefit from a mixed-methods approach, incorporating qualitative interviews to capture subjective experiences alongside psychological assessments to measure mental health outcomes more systematically. Such an approach would offer a comprehensive perspective on how family dynamics shape emotional wellbeing and could inform the development of tailored interventions.

Further, given the cross-sectional nature of the study, adopting a longitudinal design would allow for a better understanding of how these patterns evolve over time, such as how the emotional and psychological challenges faced by siblings and parents changed as children with disabilities age. Understanding the emotional and psychological outcomes within these family systems would contribute to a more comprehensive insight into broader family dynamics and could lead to more targeted interventions to support families (Fairfax et al., 2019; Dickinson et al., 2020; Wolff et al., 2023).

## Data Availability

The raw data supporting the conclusions of this article will be made available by the authors, without undue reservation.
